# Risk and recovery among high school athletes who sustained at least one sports-related concussion

**DOI:** 10.2217/cnc-2020-0002

**Published:** 2020-04-09

**Authors:** Adam R Kinney, Dustin Anderson, Kelly A Stearns-Yoder, Lisa A Brenner, Jeri E Forster

**Affiliations:** 1 Rocky Mountain Mental Illness Research Education and Clinical Center (MIRECC), Department of Veterans Affairs, Aurora, Colorado; Department of Physical Medicine & Rehabilitation, University of Colorado Anschutz Medical Campus, Aurora, CO 80045, USA; 2 Department of Physical Medicine & Rehabilitation, University of Colorado Anschutz Medical Campus, Aurora, CO 80045, USA; 3 Rocky Mountain MIRECC, Department of Veterans Affairs, Aurora, Colorado; Departments of Physical Medicine & Rehabilitation, Psychiatry and Neurology, University of Colorado Anschutz Medical Campus, Aurora, CO 80045, USA

**Keywords:** athletes, concussion, high school, mild traumatic brain injury, recovery, risk

## Abstract

**Aim:**

Evidence of factors explaining sports-related concussion (SRC) risk and recovery among high school athletes remains inconclusive.

**Materials & methods:**

Prospective study of a real-world sample of high school athletes (n = 77) who sustained ≤1 SRC. Among those with multiple SRCs, recovery time between events was investigated. To investigate concussion risk, baseline characteristics of athletes with a single versus multiple SRC(s) were compared.

**Results:**

Recovery time did not differ across events. There were no differences between those with a single versus multiple SRCs.

**Conclusion:**

Recovery time between initial and subsequent concussive events did not differ, suggesting that prior concussion may not prolong recovery. Baseline characteristics did not explain heightened concussion risk. Investigation of these relationships using more representative samples is needed.

## Lay abstract

Evidence that prior concussions and pre-injury symptoms explain sports-related concussion (SRC) risk and recovery among high school (HS) athletes remains inconclusive. This may be due to studies investigating HS athletes in medical settings. These athletes may experience more severe SRC, and therefore may not represent risk and recovery in real-world settings. We used a real-world sample of HS athletes to investigate whether prior concussion prolongs recovery for subsequent concussive events. To investigate heightened risk for concussion during the study period, we also examined differences between athletes who sustained a single versus multiple SRC during the study period. There were no differences between those with single versus multiple SRC(s). Based on our real-world sample of HS athletes, recovery time across concussive events during the study period did not differ, suggesting that a prior concussion may not prolong recovery.

A sports-related concussion (SRC) is a subtype of mild traumatic brain injury that is related to participation in athletics and is ‘induced by biomechanical forces’ [1]. An estimated 1.6–3.8 million SRCs occur in the USA each year [1]. Concussions are associated with a constellation of somatic (e.g., headache), neurocognitive (e.g., poor concentration) and emotional/behavioral (e.g., irritability) symptoms [2], which may undermine an athlete’s quality of life [3].

Evidence indicates that SRCs among high school (HS) athletes may have unique features compared with those sustained by members of other populations. SRCs among HS athletes occur relatively frequently, with an estimated incidence of 24 per 1000 [4]. Findings from a large-scale prospective study indicated that, compared with collegiate football players, HS football players had a greater risk of developing SRC [5]. Further, a recent systematic review [6] revealed that HS athletes may be particularly vulnerable to a prolonged recovery from a SRC. Younger athletes may have heightened vulnerability to SRC relative to other populations due to lower skill levels and a tendency towards more playing time [5]. Nonetheless, given the prevalence of SRC in HS athletes, along with their distinct recovery trajectory, the clinical management of concussion within HS athletes would benefit from further study of factors associated with their concussion risk and recovery [6].

Correlates of enhanced risk for and prolonged recovery from SRC have been the target of considerable scientific investigation. Presence of a prior concussion and pre-injury symptoms, such as a headache, have been linked to increased risk for [7] and prolonged recovery from [6] SRC. However, evidence that prior concussions and pre-injury symptoms explains concussion risk and recovery remains relatively inconclusive [6,7]. Mixed evidence concerning factors associated with concussion risk and recovery may be explained by methodological limitations. For example, investigations of SRC that are relevant to HS athletes contain significant methodological limitations [8], including retrospective designs and studies conducted in medical settings [9].

Dise-Lewis *et al.* [9] advanced our understanding of risk for and recovery from SRC in HS athletes, while avoiding the above methodological limitations. The present study is a secondary analysis of their prospective longitudinal study of HS athletes. Importantly, the present sample of HS athletes was derived from a real-world setting, rather than a medical setting. Samples of athletes seeking medical care may represent a group experiencing heightened injury severity [6], thereby producing findings that are not necessarily applicable to the general population of concussed athletes.

While previous studies have investigated risk and recovery in HS athletes using samples derived from real-world settings [10,11], they possess notable limitations that our study addresses. First, Kerr *et al.* [11] did not include a measure of symptom severity, instead measuring full recovery as the absence of symptoms, irrespective of severity. As lifestyle factors such as stress may confound symptom reporting among athletes postconcussion [6], this study addresses this limitation by defining recovery in terms of return to a baseline level of symptom severity. Second, the unit of analysis in Chrisman *et al.* [10] was concussions, rather than the individual, thereby precluding the comparison of recovery time between individuals and across events. This study addresses this limitation by comparing recovery time within the same individual across initial and subsequent concussive events, allowing us to glean whether recent concussion history influences recovery time from SRC.

The purpose of this study was to increase understanding regarding SRC risk and recovery among HS athletes. A particular area of interest included a comparison of time to recovery across concussive events among those who experienced multiple SRCs during the study period. Moreover, to investigate heightened risk for SRC during the study period, we sought to understand potential baseline differences (e.g., concussion history and pre-injury symptoms) between those who sustained a single SRC versus those who sustained multiple SRCs.

## Materials & methods

### Research design

Complete details of the study design can be found in the original study [9]. Briefly, this was a prospective longitudinal study that investigated pre and post-injury Immediate Postconcussion Assessment and Cognitive Testing (ImPACT) performance over a 3-year study period. The original sample consisted of 914 HS athletes (grades 9–12) attending a large public HS in suburban Colorado (USA). The present study will consider the 77 students (8.4%) who sustained concussion(s) during play or practice, as determined by the athletic trainer at the time of event. Following the concussion, the ImPACT was readministered as soon as possible (median = 3, range = 1–13 days). ImPACT scores were gathered approximately every week for 8 weeks, or until return to baseline was achieved (see below). Data collection was co-ordinated by the school psychologist. This study received approval by the Colorado Multiple Institutional Review Board.

### Measures

#### Presence of concussion

A SRC was defined as “*a blow to the head, followed by altered mental status, as assessed by the athletic trainer present at the sports event or corroborated by the student athletic trainer (under the supervision of the athletic trainer)”* [9].

#### ImPACT

The ImPACT [12], a computerized neuropsychological test, was used to measure severity of postconcussive symptoms, such as somatic symptoms, as well as four aspects of neurocognitive function: verbal memory, visual memory, visual motor speed and reaction time. We also used the ImPACT to collect demographic data, including concussion history.

#### Time to recovery

Recovery was defined as scores returning to baseline levels (within the standard error of measurement) on three out of four ImPACT neurocognitive indices (verbal memory, visual memory, motor testing and reaction time) [13]. Time to recovery was the number of days between the injury event and ImPACT testing that achieved the above recovery criteria.

#### Data analyses

All analyses were performed using SAS v9.4. Among those with multiple SRCs, we compared the time to recovery for the first and second concussions during the study period using a signed-rank test [14]. Additionally, we examined postconcussive symptom totals and cognitive function scores across the first and second concussions using descriptive statistics (i.e., median and range). We compared athletes with one SRC versus those who sustained multiple SRCs with respect to baseline (i.e., preseason) categorical (e.g., presence of a prior concussion) and continuous (e.g., postconcussive symptoms) characteristics using Fisher’s exact and Wilcoxon rank-sum tests, respectively [14].

## Results

Of the 77 athletes included in this study, 68 (88.3%) sustained one concussion during the 3-year study period; nine (11.7%) sustained two or more. One of these nine athletes sustained three concussions during the study period. To investigate heightened risk for SRC during the study period, we compared those with single versus multiple SRC with respect to baseline characteristics. At baseline, those with single versus multiple SRCs during the study period did not significantly differ with respect to age, gender, concussion history or ImPACT testing (Table 1).

**Table 1.** Baseline characteristics of individuals in single and multiple sports-related concussion groups.

	One concussion (N = 68)	Multiple concussions (N = 9)	p-value^†^
Age	14.6 (1.0) 14 (13–18)	14.4 (1.0) 14 (13–16)	0.58
Male gender	48 (71%)	5 (56%)	0.44
Grade			0.86
9	43 (63%)	6 (67%)	
10	13 (19%)	1 (11%)	
11	8 (12%)	1 (11%)	
12	4 (6%)	1 (11%)	
Prior concussion	17 (25%)	2 (29%)	<0.99
Time to 1st concussion (days)	195.8 (231.7) 60 (1–870)	108.8 (132.4) 51 (7–428)	0.53
Baseline symptom total	9.37 (11.9) 5 (0–69)	9.89 (8.4) 9 (0–30)	0.47
Baseline verbal memory	0.86 (0.09) 0.87 (0.63–1.0)	0.84 (0.13) 0.89 (0.58–0.96)	0.92
Baseline visual memory	0.79 (0.11) 0.79 (0.45–0.95)	0.74 (0.13) 0.75 (0.53–0.96)	0.25
Baseline motor	34.8 (8.8) 35.2 (44–100)	38.8 (5.9) 38.0 (31.4–48.7)	0.25
Baseline reaction time^‡^	0.56 (0.08) 0.55 (0.29–0.75)	0.55 (0.07) 0.53 (0.45–0.65)	0.65

Mean standard deviation and median (range) or N (%) are presented.

† Continuous variables were compared using Wilcoxon rank-sum tests and categorical variables were assessed using Fisher’s exact tests.

‡ For baseline reaction time, N = 67 for the one concussion group.

Four of the athletes with multiple SRCs were female (44%) and the median age at injury was 14 (range: 13 to 16). All five of the male athletes who sustained multiple concussions played football. We compared recovery time between first and second concussions for those who achieved recovery within the 8-week evaluation period for both injury events (n = 7). There was no significant difference in recovery time between first and second concussions among the seven athletes who achieved recovery in both instances (p = 0.13). Median time to recovery post-first concussion was 8 days (range: 2 to 20), and was 3 days (range: 2 to 13) post-second concussion. Additionally, similar symptom totals and cognitive test results were observed across evaluations when comparing scores post-first concussion to post-second concussion (Table 2).

**Table 2.** Median (range) postconcussion ImPACT scores.

Test	First concussion			Second concussion			
	1st Eval N = 9	2nd Eval N = 9	3rd Eval N = 6	4th Eval N = 2	1st Eval N = 9	2nd Eval N = 6	3rd Eval N = 1
Symptom total	17 (2–83)	5 (0–68)	0.50 (0–26)	25 (1–49)	19 (4–66)	4.5 (0–8)	3
Verbal memory	0.70 (0.42–1.0)	0.75 (0.48–0.94)	0.77 (0.51–0.96)	0.62 (0.54–0.71)	0.78 (0.50–0.91)	0.94 (0.46–0.97)	0.77
Visual memory	0.59 (0.41–0.97)	0.76 (0.30–1.0)	0.70 (0.53–0.86)	0.60 (0.50–0.71)	0.74 (0.49–0.96)	0.81 (0.38–0.99)	0.67
Motor	36.0 (14.0–51.3)	33.5 (10.8–44.7)	33.8 (16.3–46.2)	36.9 (29.3–44.6)	34.3 (15.8–42.3)	41.0 (10.9–50.7)	42.3
Reaction time	0.60 (0.50–0.81)	0.50 (0.41–0.84)	0.63 (0.49–0.71)	0.60 (0.51–0.68)	0.60 (0.45–0.79)	0.60 (0.44–0.75)	0.48

Eval = Administration of ImPACT.

## Discussion

This study leveraged a sample of concussed HS athletes, obtained in a real-world setting, to examine SRC risk and recovery. Results indicate that among athletes who sustained multiple SRCs during the study period, time to recovery for initial and subsequent concussive events did not differ. Our finding that recovery time was similar across initial and subsequent concussive events contributes to an emerging body of evidence which indicates that concussion history may not be a relatively robust predictor of clinical recovery [6]. Interestingly, we also found that athletes who sustained multiple SRCs during the study period, a cohort with seemingly heightened vulnerability to concussion, did not differ from those who sustained one concussion in terms of concussion history or severity of baseline (pre-injury) postconcussive symptoms. Findings require replication with more representative samples, but nonetheless offer contributions to our understanding of concussion risk and recovery among HS athletes.

Among HS students with multiple concussions, there was no significant difference in recovery time between their first and second concussions. This finding contributes to mounting evidence that the presence of a prior concussion may be a relatively weak predictor of concussion recovery [6]. Mixed evidence for the link between concussion history and clinical recovery may be explained by methodological limitations within studies relevant to SRC among HS athletes [8]. For example, samples from medical rather than real-world settings may represent a subgroup of patients who experience more severe injuries. Given the consistency with which injury severity predicts concussion recovery, the severity of the SRC may better explain recovery time among medical samples, rather than a history of concussion [6]. Importantly, our finding that the presence of a prior concussion during the study period did not influence recovery time for the second was derived using a sample from a real-world setting, thereby enhancing its ecological validity. Future research on SRC recovery should investigate concussion history, while accounting for the influence of other factors related to recovery, such as injury severity and pre-injury mental health problems [6].

We compared the baseline characteristics of those who sustained one versus multiple SRCs during the study period to investigate potential predictors of heightened risk for concussion. Our findings indicate that those who sustained one SRC during the study period had similar concussion histories compared with those who sustained multiple SRCs. There is high-quality evidence indicating that past concussion increases risk for future concussion [7,9]; however, these findings typically emerge from prospective studies simply comparing those who were concussed during the study period to the nonconcussed. Further, these findings are often derived using samples from medical settings, which may be comprised of patients who experience atypically severe injuries [6]. To the best of our knowledge, this is the first prospective study conducted in a real-world setting that examined whether concussion history distinguished those who sustained one versus multiple concussions during the study period. While our findings should be considered preliminary, they suggest that concussion history may not explain the seemingly elevated vulnerability to concussion present among those who sustain repeated concussions. Future research should investigate factors capable of distinguishing those vulnerable to occasional versus repeated SRC. For example, Iverson *et al.* [6] highlighted severity of concussion-related symptoms as a robust predictor of clinical recovery. Studies should leverage more representative samples to investigate whether concussion history and/or other factors (e.g., severity of past concussions) explain heightened vulnerability to repeated concussions.

HS athletes in our sample who sustained multiple concussions did not have more severe baseline postconcussive symptoms and cognitive dysfunction than those who sustained one concussion. Previous research indicates that the relationship between pre-injury symptom severity and clinical recovery may be partially explained by past concussions [9]. However, there is an emerging understanding that factors independent of concussion history may be relevant. Many participants report baseline symptoms that are due to lifestyle factors such as stress or sleep disruption, thereby complicating our understanding of the link between symptom severity and concussion risk and recovery [6]. Future research should investigate the relationship between baseline symptom severity and concussion risk while controlling for potentially confounding lifestyle factors (e.g., stress).

This study includes considerable strengths, most notably its prospective design and real-world sample. Nonetheless, the study contained several limitations which require noting. First, bias may be present due to the lack of blinding and the fact that the same psychologist coordinated data collection and provided care. However, we attempted to counterbalance any bias by using computerized testing. Further, the time to evaluations was not standardized. In addition, recovery was defined as return to baseline levels of symptom reporting; while symptom reporting is a widely adopted method for defining recovery from SRC, future studies should employ objective physiological measures and self-report measures in tandem to define recovery [15]. A total of 17 athletes (25%) who sustained one SRC during the study period reported a history of concussion prior to the study. This indicates that while these 17 athletes sustained multiple concussions over their lifetime, for the purposes of this study we could not include them in the ‘multiple SRC’ cohort because we did not have the requisite data to compare their initial and subsequent recoveries. Relatedly, the small sample size limits statistical power and the generalizability of findings. As mentioned, however, the ecological validity of our findings benefits from our sample of HS athletes obtained in a real-world setting. Nonetheless, future research should replicate findings using larger, more representative samples to rule out sampling bias and Type II error. Further, future research investigating predictors of SRC risk and recovery should account for potentially confounding factors such as age and gender [6].

## Conclusion

This study advanced our understanding of factors associated with SRC risk and recovery among HS athletes. Results, which were collected outside of a medical setting, indicate that, among those with multiple SRCs, recovery time frames did not differ and thus indicate that prior concussion during the study period did not prolong recovery. Athletes who sustained multiple concussions during the study period did not differ from those who sustained one concussion in terms of concussion history or baseline symptom severity. These unexpected findings conflict with perspectives emphasizing the role of concussion history as predictive of concussion risk and clinical recovery and warrant further investigation. Most notably, prior studies often leveraged samples comprised of those who were seeking medical care, resulting in a sample with potentially heightened injury severity [16]. Our findings, derived using a real-world sample, merit replication and support future investigations of alternative predictors of concussion risk and recovery among HS athletes.

## Future perspective

As SRCs continue to capture the attention of society at large, the research community will be compelled to achieve consensus on the factors that explain SRC risk and recovery among HS athletes. To answer this charge, the research community should employ prospective studies that leverage representative samples of HS athletes within their natural settings. In such studies, researchers should examine constructs beyond concussion history and baseline symptom severity, including the severity of the concussive event and pre-injury psychiatric history. Employing such methods may allow the field to arrive at a set of risk factors that reliably predict the likelihood of, and prolonged recovery from, SRC. Consequently, the establishment of factors explaining risk and recovery from SRC may contribute to the development of effective strategies aimed at preventing and/or managing SRC.

**Summary points**

- High school (HS) athletes are vulnerable to sports-related concussion (SRC), with an estimated incidence of 24 per 1000.
- Presence of a prior concussion and pre-injury symptoms (e.g., headache) have been linked to increased risk for, and prolonged recovery from, SRC; however, the evidence remains inconclusive.
- Inconclusive evidence may be due to methodological limitations; studies often use samples of HS athletes in medical settings, who may experience more severe SRC and therefore may not represent risk and recovery in real-world settings.
- This was a prospective, longitudinal study of HS athletes in a real-world setting that investigated whether a prior concussion prolonged recovery from subsequent concussions.
- This study also compared athletes who sustained a single versus multiple SRCs, with respect to concussion history and baseline symptom severity.
- Among those with multiple SRCs, recovery timeframes did not differ, indicating that prior concussion did not prolong recovery.
- Concussion history and baseline symptoms did not distinguish athletes who sustained one versus multiple SRCs, indicating they did not enhance risk for SRC in the study period.
- Further investigation of these relationships using more representative samples is needed.

## References

[1] Langlois JA, Rutland-Brown W, Wald MM The epidemiology and impact of traumatic brain injury: a brief overview. J. Head Trauma Rehabil. 21(5), 375–378 (2006). 16983222 10.1097/00001199-200609000-00001

[2] Taylor AM Neuropsychological evaluation and management of sport-related concussion. Curr. Opin. Pediatr. 24(6), 717–723 (2012). 23080132 10.1097/MOP.0b013e32835a279b

[3] Kuehl MD, Snyder AR, Erickson SE, Mcleod TCV Impact of prior concussions on health-related quality of life in collegiate athletes. Clin. J. Sport Med. 20(2), 86–91 (2010). 10.1097/JSM.0b013e3181cf4534 20215889

[4] Lincoln AE, Caswell SV, Almquist JL, Dunn RE, Norris JB, Hinton RY Trends in concussion incidence in high school sports: a prospective 11-year study. Am. J. Sports Med. 39(5), 958–963 (2011). 21278427 10.1177/0363546510392326

[5] Guskiewicz KM, Weaver NL, Padua DA, Garrett WE Epidemiology of concussion in collegiate and high school football players. Am. J. Sports Med. 28(5), 643–650 (2000). 11032218 10.1177/03635465000280050401

[6] Iverson GL, Gardner AJ, Terry DP Predictors of clinical recovery from concussion: a systematic review. Br. J. Sports Med. 51(12), 941–948 (2017). 28566342 10.1136/bjsports-2017-097729 PMC5466929

[7] Abrahams S, Mc Fie S, Patricios J, Posthumus M, September AV Risk factors for sports concussion: an evidence-based systematic review. Br. J. Sports Med. 48(2), 91–97 (2014). 24052371 10.1136/bjsports-2013-092734

[8] Spiotta AM, Shin JH, Bartsch AJ, Benzel EC Subconcussive impact in sports: a new era of awareness. World Neurosurg. 75(2), 175–178 (2011). 21492686 10.1016/j.wneu.2011.01.019

[9] Dise-Lewis JE, Forster JE, Mcavoy K The natural history of postconcussion recovery among high school athletes. J. Head Trauma Rehabil. 34(5), E36–E44 (2019). 30829818 10.1097/HTR.0000000000000469

[10] Chrisman SP, Rivara FP, Schiff MA, Zhou C, Comstock RD Risk factors for concussive symptoms 1 week or longer in high school athletes. Brain Inj. 27(1), 1–9 (2013). 23252433 10.3109/02699052.2012.722251

[11] Kerr ZY, Zuckerman SL, Wasserman EB Factors associated with post-concussion syndrome in high school student-athletes. J. Sci. Med. Sport 21(5), 447–452 (2018). 28939003 10.1016/j.jsams.2017.08.025

[12] Grindel SH, Lovell MR, Collins MW The assessment of sport-related concussion: the evidence behind neuropsychological testing and management. Clin. J. Sport Med. 11(3), 134–143 (2001). 11495317 10.1097/00042752-200107000-00003

[13] Manderino LM, Gunstad J Performance of the immediate post-concussion assessment and cognitive testing protocol validity indices. Arch. Clin. Neuropsychol. 33(5), 596–605 (2017). 10.1093/arclin/acx102 29088321

[14] Field A, Miles J, Field Z Discovering Statistics Using R. SAGE Publications, CA, USA (2012).

[15] Haider MN, Leddy JJ, Pavlesen S A systematic review of criteria used to define recovery from sport-related concussion in youth athletes. Br. J. Sports Med. 52(18), 1179–1190 (2018). 28735282 10.1136/bjsports-2016-096551 PMC5818323

[16] Dise-Lewis JE, Lewis HC, Reichardt CS BrainSTARS: pilot data on a team-based intervention program for students who have acquired brain injury. J. Head Trauma Rehabil. 24(3), 166–177 (2009). 19461364 10.1097/HTR.0b013e3181a7ecb0

